# Brain-Body Control of Glucose Homeostasis—Insights From Model Organisms

**DOI:** 10.3389/fendo.2021.662769

**Published:** 2021-03-31

**Authors:** Alastair J. MacDonald, Yu Hsuan Carol Yang, Ana Miguel Cruz, Craig Beall, Kate L. J. Ellacott

**Affiliations:** Institute of Biomedical and Clinical Sciences, University of Exeter Medical School, Exeter, United Kingdom

**Keywords:** brain, glucose homeostasis, model organism, mouse, rat, zebrafish

## Abstract

Tight regulation of blood glucose is essential for long term health. Blood glucose levels are defended by the correct function of, and communication between, internal organs including the gastrointestinal tract, pancreas, liver, and brain. Critically, the brain is sensitive to acute changes in blood glucose level and can modulate peripheral processes to defend against these deviations. In this mini-review we highlight select key findings showcasing the utility, strengths, and limitations of model organisms to study brain-body interactions that sense and control blood glucose levels. First, we discuss the large platform of genetic tools available to investigators studying mice and how this field may yet reveal new modes of communication between peripheral organs and the brain. Second, we discuss how rats, by virtue of their size, have unique advantages for the study of CNS control of glucose homeostasis and note that they may more closely model some aspects of human (patho)physiology. Third, we discuss the nascent field of studying the CNS control of blood glucose in the zebrafish which permits ease of genetic modification, large-scale measurements of neural activity and live imaging in addition to high-throughput screening. Finally, we briefly discuss glucose homeostasis in drosophila, which have a distinct physiology and glucoregulatory systems to vertebrates.

## Introduction

The central nervous system has emerged as an important node in the coordinated control of blood glucose homeostasis. Maintenance of euglycemia is critical for health and the regulation of energy homeostasis. Extended periods of poor glycemic control drive disease pathology; for example, prolonged hyperglycemia in type-1 and type-2 diabetes can cause eye, kidney, and nerve damage over the longer term. Conversely, low blood glucose or hypoglycemia is also dangerous acutely, with recurrent bouts leading to deficits in hypoglycemia awareness, which can result in death in extreme but rare circumstances. Given this importance, in healthy individuals, highly sensitive feedback systems exist to regulate blood glucose within a tight window. Appropriate regulation of blood glucose is ultimately dependent on the correct balance between glucose ingestion, production, utilization, and storage. This control is achieved by communication between multiple organ systems, chiefly the gastrointestinal tract, pancreas, muscle, liver, adipose, adrenal glands, and brain.

When combined with classical physiological approaches, modern genetic manipulation technologies have refined our understanding of the critical role of the brain in overseeing glucose homeostasis and orchestrating appropriate physiological and behavioral responses (1, 2). In addition to well described endocrine inter-organ communication, cell populations in discrete brain nuclei are sensitive to acute deviations in tissue glucose levels and some are also capable of direct glucose sensing (3–6). Furthermore, sensory innervation of organs relays relevant information on peripheral glucose state to the brain (7, 8). By altering autonomic outflow, the brain drives responses to these deviations in blood glucose, helping to restore homeostasis (9, 10).

**Figure 1:**
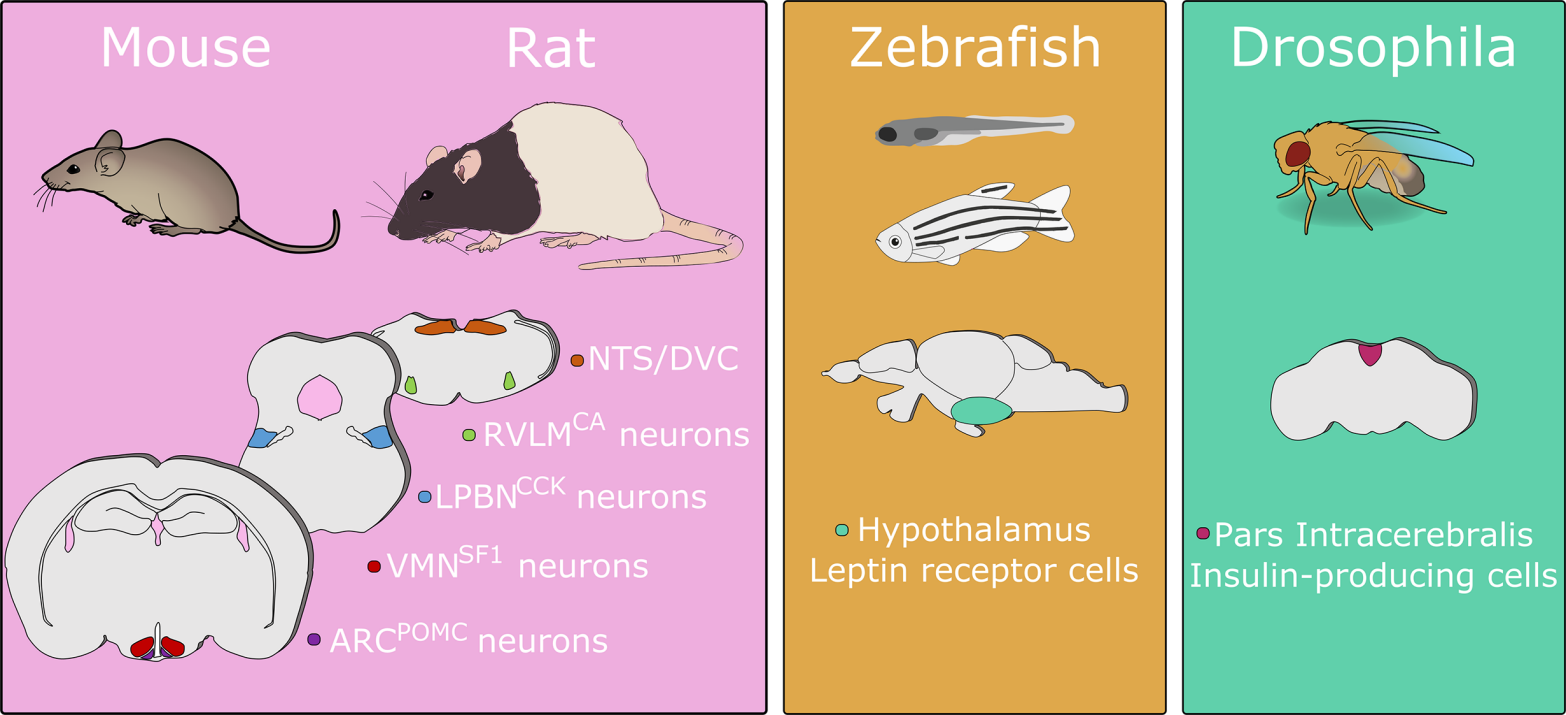
A simplified overview of primary brain sites regulating blood glucose identified in model organisms. This figure shows the cell populations discussed in this review and is not an exhaustive list. Numerous brain regions have been demonstrated to contribute to glucose regulation in mice and/or rats. In zebrafish disruption of leptin receptor signaling induces hyperglycemia. In drosophila, ablation of insulin-producing cells causes increases in hemolymph carbohydrate levels. Abbreviations: ARC, arcuate nucleus of the hypothalamus; CA, catecholamine; CCK, cholecystokinin; DVC, dorsal vagal complex; LPBN, lateral parabrachial nucleus; NTS, nucleus of the solitary tract; POMC, pro-opiomelanocortin; RVLM, rostral ventrolateral medulla; SF1, steroidogenic factor 1; VMN, ventromedial nucleus of the hypothalamus. Animal images adapted from Scidraw.io. Drawings not to scale.

Much of what is known about crosstalk between the body and brain is the result of experimentation in model species. A simplified overview of the main brain regions and cell populations identified in blood glucose control in common model species is shown in **Figure 1**. Comprehensive reviews on both the different species used to model human metabolic disease and descriptions of glucoregulatory neurocircuitry are available elsewhere (11–13). Instead, in this mini-review, using select examples from the literature, we will focus on the utility of different model organisms to specifically elucidate neural circuits regulating glucose homeostasis.

### Mice

Mice have emerged as the most commonly used rodent species for neuroscience research (14). This is due, at least in part, to the suite of transgenic tools available. Furthermore, mice are amenable to measures of systemic glucose homeostasis, commonly glucose and insulin tolerance tests, but glucose clamps are also possible (15–17).

The combination of genetic tools and means for real-time measurement of changes in systemic glucose levels in mice permits the investigation of the role of the brain in control of blood glucose homeostasis. The ventromedial nucleus of the hypothalamus (VMN) has long been implicated in the neural control of energy balance and blood glucose levels (3, 4, 18). Cre-lox recombination has been used to elegantly dissect the role of a defined neuronal population within this nucleus in glucose homeostasis: using a driver line expressing Cre recombinase in the VMN (Steroidogenic factor 1-Cre) to knock out vesicular glutamate transporter 2 selectively in VMN neurons (19). These mice lack VMN glutamatergic transmission and have impaired sensitivity to fasting, insulin and 2-deoxyglucose (2-DG). Taken together, this shows that glutamatergic transmission in VMN neurons is an essential component for the counter-regulatory response to hypoglycemia (CRR) (19). Thus, this approach can be used to generate causal evidence of a specific process (glutamatergic neurotransmission), in a defined brain area (VMN) required for a physiological process (CRR). Similarly, Cre-lox recombination can be used to selectively re-express a gene in genetically defined cells in knockout animals (20, 21). This permits testing of both necessity (knock-out) and sufficiency (selective re-expression) of a gene of interest in a specific cell population.

This recombination method can be refined with drug-inducible forms of Cre (e.g. tamoxifen inducible cre; CreERT2). The importance of temporal control of recombination is illustrated in the case of leptin receptor (LepR) expression on pro-opiomelanocortin cells (POMC cells). Embryonic deletion of *LepR* from POMC cells results in mice that have a greater body and fat mass than control animals with intact leptin signaling in POMC cells (22). However in adult mice where *LepR* is knocked out of adult POMC cells by providing tamoxifen to POMC^CreERT2^ mice, this phenotype is absent (23). Instead, these mice show a hyperglycemic phenotype driven by increased hepatic glucose production and insulin resistance, while body weight and energy expenditure are normal (23). This reveals a glucoregulatory role of LepR signaling in POMC cells in adult mice which may have been masked by the embryonic knockout and thus highlights potential developmental compensation as a caveat to Cre recombination without temporal control. Furthermore, it has been shown that a number of ventral hypothalamic cells transiently express POMC during development but not in adulthood, including functionally opposed agouti-related peptide (AgRP) expressing neurons (24). Thus, by using temporally restricted Cre-recombination, manipulations can be limited to cells of interest in adulthood rather than all cells derived from a POMC-expressing lineage.

In addition to the selective embryonic genetic (or inducible adult) knockout models described above, mice are also amenable for the selective stimulation or inhibition of defined neuronal populations in adult animals by optogenetic or chemogenetic methods (25, 26). For example, using chemogenetics, stimulation of neurons in the lateral parabrachial nucleus (LPBN) identified by their expression of cholecystokinin (CCK; LBPN^CCK^ neurons) causes a CCK-dependent increase in blood glucose driven by increased plasma glucagon, corticosterone and epinephrine levels (27). Chemogenetic inhibition of this neuronal population leads to an attenuated blood glucose increase in response to 2-DG-induced glucoprivation. Using this methodology to selectively inhibit the VMN neurons, identified by their expression of steroidogenic factor 1 (SF1; VMN^SF1^ neurons), while stimulating upstream LPBN^CCK^ neurons occludes the rise in blood glucose induced by this stimulation. This suggests that LPBN^CCK^ neurons are involved in hypoglycemia detection and relay this information to VMN^SF1^ neurons to exert compensatory changes in blood glucose. This illustrates the power of experimental tools which enable bi-directional modulation of neuronal populations of interest (specific activation or inhibition) while measuring physiological parameters. In addition, it highlights how concomitant manipulation of pre- and post-synaptic neurons, respectively, can demonstrate the necessity of a given projection site for the observed effects. The same viral approach can be used to drive expression of fluorescent proteins in genetically defined cell populations. This expands the scope of this method to tracing anatomical circuits in addition to probing their function. These tools also have the advantage of being specific and targetable in adult animals, avoiding the potential developmental adaptations and complications associated with embryonic deletion of genes.

In recent years, studies in mice have characterized sensory neurons of the vagus nerves and their role in relaying information on internal state from the periphery to the brain (28–33) including cardiovascular, pulmonary, and gastrointestinal parameters. These signals appear to exist to drive appropriate autonomic responses (i.e., vago-vagal reflexes) in addition to modulating associated behavior. For example, stimulation of gut-innervating vagal sensory neurons elicits changes in gastric motility and pressure while also suppressing food intake (28, 31, 33). Given this evidence, the existence of a vagal sensory circuit monitoring blood glucose, communicating this to the brain and driving both appropriate autonomic and behavioral responses seems possible. Vagal sensory innervation of the liver and pancreas have been described in classical studies performed in rats (34, 35). With the platform already developed for functional and genetic investigation of vagal sensory subtypes (28–32), this represents an area ripe for investigation using contemporary neuroscience techniques. However, a recent report suggests that the detection of ingested glucose by hypothalamic neurons is independent of the vagus nerve, instead this signal is relayed by spinal afferents monitoring the hepatic portal vein (36).

In complementary studies, vagal efferent pathways regulating blood glucose have been examined in mice. Vagal sensory neurons from peripheral organs terminate in the nucleus of the solitary tract (NTS), while vagal efferent neurons originate in the neighboring dorsal motor nucleus of the vagus (DMV). Vagal efferent neurons of the DMV are identified by expression of choline acetyltransferase (ChAT; DMV^ChAT^ neurons). These DMV neurons are innervated by NTS neurons, providing an anatomical substrate for vago-vagal processes. Acute chemogenetic activation of inhibitory NTS neurons (NTS^GABA^ neurons (10)), which receive direct vagal input (37), increases blood glucose by reducing the tonic activity of DMV^ChAT^ neurons. This disinhibits hepatic glucose production (10). Thus, the cellular architecture of vago-vagal signaling loops are beginning to be elucidated in mice.

As described above, contemporary neuroscience technologies allow for the selective manipulation of activity and/or gene expression of spatially and genetically defined cell types, both in the brain and peripheral ganglia. Combined with standard tests of glucose homeostasis and the similarities between mouse and human physiology (11), this presents a powerful platform on which to examine neural circuits governing blood glucose homeostasis. However, this species and these approaches are not without their caveats. Strains and sub-strains of inbred mice have demonstrably different responses to modulation of blood glucose both in terms of glucose-stimulated insulin secretion and glucoprivic feeding (38–40). Some of these differences arise from known genetic mutations, for example the nicotinamide nucleotide transhydrogenase mutation in the C57Bl6/J sub-strain (41). As such, outbred strains may be more suitable for some experiments to reduce the impact of single mutations on observed measurements (38, 41).

Care should also be taken with respect to studies utilizing Cre-lox recombination. Mice expressing Cre recombinase can have phenotypes arising from off target recombination or integration of Cre into a functional gene (42). As such, it is important to consider the appropriate control groups, including littermates, to account for this [discussed in detail in (33)]. In addition, embryonic Cre recombination or transient expression in off target tissues may account for a phenotype in adult animals that does not represent the function of the gene in adult physiology. Finally, opto- or chemogenetic manipulations may induce artificial activity patterns that demonstrate the consequence of cellular activation, but should be interpreted with caution as may not reflect the “normal” function of those cells. Moreover, with chemogenetic experiments, it is important to control for both off target effects of chemogenetic ligands and expression of the receptor which may have constitutive activity in the cell type of interest (43, 44). These limitations and key experimental controls also apply to related studies in transgenic rats (see below).

### Rats

Rats offer some major advantages over mice, such as their larger size and the fact that many common strains (i.e., Sprague Dawley, Wistar) are outbred, increasing the generalizability of the data for human populations. This outbred nature can increase variability in a study and combined with the size can make studies more expensive. However, the greater size allows for collection of larger blood sample volumes, which can be advantageous for endocrine studies. For the advanced assessment of glucose homeostasis, it can be beneficial to surgically implant indwelling vascular catheters in a vein and/or an artery (i.e., jugular vein and carotid artery). This enables repeated sampling with a continuous infusion in conscious, freely moving rats, which is useful during hyperinsulinemic glucose clamping (45). However, protocols for clamping in mice are highly refined (46). From a rat, the larger blood samples volumes can be taken without the need for concomitant replacement of blood from donor animals (although blood cells can be re-suspended and re-infused during a clamp). Moreover, recent advances in indwelling vascular access buttons (VAB) have allowed for streamlined blood sampling, maintenance of catheter patency, and attachment of animals to the glucose clamping apparatus. This is advantageous over harnesses, which can cause chaffing as the rat moves and/or grows. These buttons also permit social housing immediately post-surgery if aluminum VAB caps are used. This may improve welfare by reducing post-surgical weight loss and improve overall recovery. This technology has also been adapted for use in mice, with some minor modifications.

Another recent technological advancement is the development of fully implantable glucose telemetry devices for continuous glucose monitoring in freely moving unrestrained rats (47). This permits glucose monitoring for up to 75 days and requires implantation of a ~2g device with a sensor tip placed in an artery. It should be noted that glucose measurements can differ between tail vein and arterial glucose, depending on the model of choice (48). These devices can also be adapted for use in mice; however, the cost of the telemetry devices and the advanced surgical procedures required have prevented widespread adoption of this technology, which is only likely to be beneficial for chronic studies.

The rat is a particularly useful model to study the neuroendocrine regulation of the CRR to acute and recurrent hypoglycemia, particularly the Sprague-Dawley and Wistar rats, which have been the workhorse of the hypoglycemia field for the last 30 years. The hyperinsulinemic-hypoglycemic clamp, together with insulin-induced hypoglycemia and 2-DG induced glucoprivation, have been extensively used in Sprague-Dawley rats to investigate CRR, glucoprivic feeding and impaired awareness of hypoglycemia (49–56). This latter aspect of hypoglycemia “awareness” can be studied using a conditioned place preference test, which has so far been validated in rats but not in mice (57), largely because of the more rapid induction of defective counter-regulation in rats and their tractability for behavioral tests. The species differences between rats and mice in the adaptation to recurrent hypoglycemia are important considerations when designing a study and have been previously discussed in detail elsewhere (58). Seminal studies in rats revealed the presence of glucosensors in the hindbrain that can mount responses to restore blood glucose in the face of a glucoprivic challenge independent of forebrain structures or when the cerebral aqueduct is blocked (59, 60). Secondly, chemical lesion studies of hypothalamus-projecting catecholaminergic neurons implicates these cells as a class of neurons underlying glucoprivic feeding (61). However, evidence suggests that hindbrain-limited recurrent glucoprivation does not alter CRR (62), suggesting that adaptations to hypoglycemia that cause defective CRR are likely forebrain-mediated. Studies by the Levin lab mapped expression of the key glucosensor, glucokinase (GK), to neurons that exhibited both glucose sensing and non-glucose sensing properties (63). In support of the physiological data described above, they also noted a relative lack of GK expression in the NTS, despite neurons in this region playing a key role in neuroendocrine and behavioral responses to hypoglycemia (64). Other key components of glucosensing originally described in the beta-cell have also been shown to play a role in hypoglycemia detection in rats. For example, pharmacological and genetic manipulation of VMN ATP-sensitive potassium channels (K_ATP_) (65) or AMP-activated protein kinase activity (66, 67) can alter CRR in healthy, recurrently hypoglycemic and diabetic rats.

With the advent of genetic technologies and the ease of applying these approaches to mice, rats have been somewhat side-lined in neuroscientific research (14). However, the first Cre-driver rat lines were described a decade ago and many more are now available (68, 69), including some which have been used to demonstrate control of blood glucose by defined neuronal populations (70). In a pair of recent studies, chemogenetic receptors were expressed selectively in anatomically distinct subgroups of catecholaminergic neurons (identified by expression of tyrosine hydroxylase [TH]) in the rat ventrolateral medulla (VLM^TH^ neurons). This was achieved by injection of Cre-dependent viral vectors into TH-Cre rats (68, 70, 71). Selective activation of these VLM-neuronal subgroups (distributed along the rostro-caudal axis) differentially increased food intake and corticosterone levels, but only in combination was activation sufficient to increase blood glucose (70). This anatomically precise modular viral transduction was facilitated by the larger size of the rat brain relative to the mouse; in a comparable study using similar methodology in dopamine-beta-hydroxylase-Cre mice the whole VLM was transduced and chemogenetic activation increased blood glucose (72). In a follow up study, again in TH-Cre rats, the same group showed that repeated glucoprivation by daily injection with 2-DG reduced the effects of chemogenetic stimulation of VLM^TH^ neurons on food intake and corticosterone release. Importantly, repeated chemogenetic activation of these neurons blunted food intake and the corticosterone response to a single bout of glucoprivation, suggesting that prior activation of this neural circuit by any means, is sufficient to blunt subsequent activation (71).

### Zebrafish

Vertebrate genetics, embryonic development, and metabolic diseases can be modelled in zebrafish (73, 74), given the well conserved organ systems, lipid metabolism, hormone secretion, and glucose homeostasis (75–78). Indeed, zebrafish are emerging as a complementary model to understand analysis of glucoregulatory organs, like the liver (79–81), muscle (82, 83), adipose (84, 85), and pancreas (86, 87). Their small size and high fecundity also make them suitable for compound screening (88) for regulators of beta cell mass and metabolism (89–97). Additionally, the major brain regions, while morphologically different, are well conserved in zebrafish (98, 99). Notably, the small size and optical transparency of larval zebrafish allows for *in vivo* activity recording of all neurons (100–102), and subsequent mapping of their anatomical brain regions (103, 104). Analogous neural circuits governing animal behavior can be found in zebrafish; including, the feeding and sleep/wake cycle associated neurotensin and hypocretin/orexin secreting neurons in the hypothalamus (105), the stress-associated hypothalamic-pituitary-adrenal axis (106), and learning and memory centers (107, 108).

While zebrafish behavioral research has been prolific, there have been few studies on the neural regulation of glucose homeostasis. Nonetheless, as in mammals, both leptin receptor and the central melanocortin systems are present in the zebrafish hypothalamus (109–111). The role of leptin in the maintenance of energy homeostasis is well studied in mammalian models (112, 113). Leptin receptor knockout (*lepr* ^-/-^) zebrafish display altered glucose homeostasis, increased beta cell mass, but normal adiposity, feeding and fertility, highlighting potentially important differences in the function of this hormone in zebrafish compared to mammals (109). Two leptin genes (*lepa* and *lepb*) exist in zebrafish. In contrast to *lepr* ^-/-^ zebrafish, *lepa* ^-/-^ zebrafish display hyperglycemia, mild obesity, increased appetite, and decreased aggression (114). Therefore, it remains to be determined whether loss of leptin signaling in zebrafish can fully recapitulate mammalian phenotypes.

In the peripheral nervous system, recent studies have demonstrated the innervation of zebrafish pancreas early in development (115, 116). Our understanding of the neural regulation of glucoregulatory organs and the respective sensory feedback loops will be advanced by future studies in zebrafish combining optogenetic (117) and chemogenetic (43) approaches to control neural signaling and detailed *in vivo* analysis of the target organ of interest on the single cell level. Targeting the desired neural populations in zebrafish will be guided by topographic mapping, which has been elegantly studied for the vagus motor nucleus in the hindbrain (118–120), and retrograde neural tracing (121). Additionally, the GAL4-Upstream Activating Sequence (UAS) system provides a flexible toolbox for driving the expression of a range of transgenes in zebrafish in a tissue specific manner (122). Changes in glucose homeostasis could also be investigated in larval and adult zebrafish. Tracking changes in circulating glucose levels within the same animal remains difficult; however, due to the relatively low housing costs and high fecundity, glucose tolerance tests could be conducted in zebrafish by sampling different animals at various time points following exposure to a glucose bolus (75). Zebrafish could provide new insights in brain-body communication, especially on a single cell level, with live animal studies that are difficult to achieve in other vertebrate models.

### Drosophila

Despite stark differences in physiology, namely the absence of an organ equivalent to the pancreas and the predominant circulation of a non-reducing sugar trehalose instead of glucose, the invertebrate *Drosophila* has been used as a model organism to study diabetes (123). Crucially, these flies can distinguish nutritive sugars (e.g. *D*-glucose) from non-nutritive sugars (e.g. *L*-glucose) independent of taste, indicating the existence of glucosensing mechanisms (124). Of interest to readers of this review, the drosophila brain contains a population of insulin-producing cells proposed to be functional equivalents of pancreatic beta cells in other species (125). When these cells are ablated in drosophila larvae the predominant phenotype is reduced growth. However, these larvae also show elevated carbohydrate (combined trehalose and glucose) levels (125). Ablation of these insulin-producing cells (IPCs) in adult flies increases hemolymph glucose levels in addition to other phenotypes including longer lifespan and stress resistance (126). These cells also share signal transduction mechanisms with mammalian beta cells including excitability increased by glucose and/or closure of the K_ATP_ channel (127, 128). This suggests that the brain may be the principal glucoregulatory site in drosophila although the functional importance of this to insect physiology is debated (123). The brain is not the sole site of glucose regulation in the drosophila however, since the drosophila equivalent of glucagon is produced by a group of neuroendocrine cells in the corpora cardiaca ([CC] analogous to the mammalian pituitary) (129). It was recently shown that both IPCs and CC cells are regulated by a pair of glucose excited neurons in the dorsolateral portion of the drosophila brain (130). These neurons, identified by co-incident expression of corazonin and short neuropeptide F, project to both IPCs and CC cells to stimulate insulin secretion and suppress glucagon secretion respectively (130).

Drosophila offer a low-cost high-throughput screening platform for disease-related genes. A recent relevant example is the description of a suite of behavioral assays in drosophila where neural expression of genes associated with appetite regulation, identified from genome wide association studies [GWAS], were disrupted (131). With available GWAS data, large-scale reverse genetic studies could potentially be adapted in drosophila to study genes regulating blood glucose (132).

To summarize, drosophila have distinct physiology from mammalian species and, while there is evidence for glucoregulatory neurocircuitry, it appears that these circuits are distinct from those in mammals, instead, resembling something closer to pancreatic cell types. This may preclude the use of drosophila to provide meaningful insight specifically into the control of glucose homeostasis by the brain but could be a useful reverse genetic screening tool for genes that impact systemic glucose homeostasis.

### Experimental Models of Diabetes

**Figure 2:**
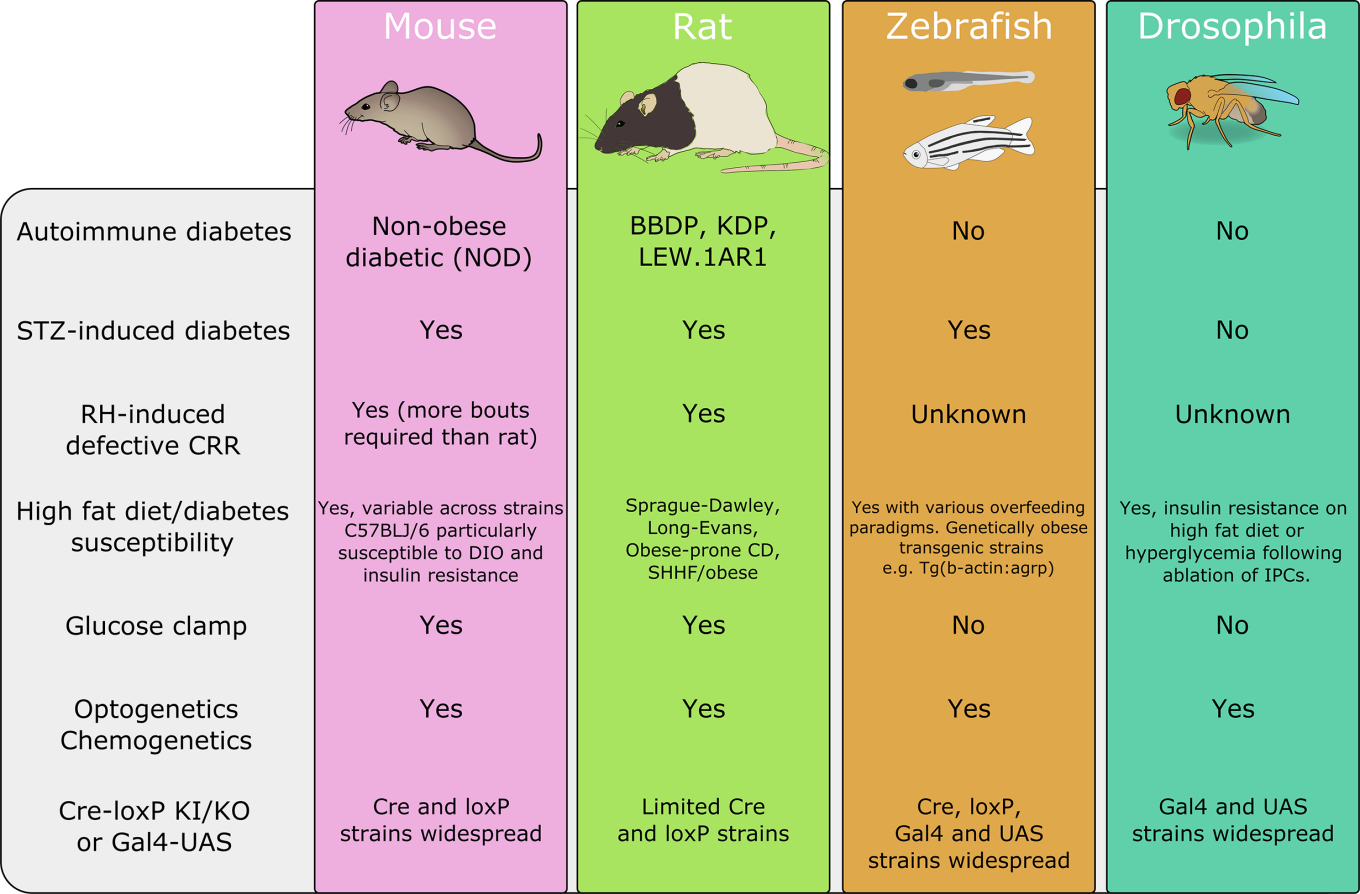
A summary of disease models and techniques available in each of the discussed organisms. BBDP, biobreeding diabetes-prone; DIO, diet-induced obesity; IPC, insulin-producing cell; KDP, Komeda diabetes prone; SHHF, spontaneously hypertensive hear failure; UAS, upstream activation sequence. This is a summary and is not intended as an exhaustive list. Animal images adapted from Scidraw.io.

While it is beyond the scope of this review to discuss in great detail the strengths and weaknesses of these model organisms to recapitulate features of human diabetes (11) we can briefly outline these here (summarized in **Figure 2**). A range of mouse and rat strains with known, spontaneous, mutations are available each with distinct phenotypes that model type 1 or 2 diabetes, with and without obesity. Complementary to these lines, disease states can be induced by feeding with a high-fat, high-sugar diet, injection with streptozotocin or repeated bouts of hypoglycemia. The effectiveness of these protocols varies between mice and rats (and within strains of these species) with C57BLJ/6 mice being especially prone to diet-induced obesity (DIO) while inbred rat strains show both resistance or susceptibility to DIO (133, 134). Similarly, rats more readily develop impaired awareness of hypoglycemia with fewer hypoglycemic bouts required for induction than mice (58). Non-mammalian species including zebrafish and drosophila also have transgenic strains that can model some facets of human disease, however, their distinct physiology from humans means that these models do not recapitulate human disease as faithfully as rodent models (135, 136).

## Conclusion

A wide variety of approaches in diverse model species has begun to identify pathways by which the brain communicates with peripheral systems to control glucose homeostasis. The strengths and weaknesses of these models are summarized in **Figure 2**. The toolbox for manipulation and monitoring of genetically defined cell types in rodents affords the ability to characterize neural circuits in a high degree of detail. However, these techniques are not without their caveats and careful experimentation and selection of control groups is required (42, 137). The hyperinsulinemic-euglycemic clamp remains the gold standard technique for assessing whole-body insulin sensitivity *in vivo* (138). Not only is vascular catheterization less technically challenging in the rat compared to the mouse, but mice require infusion of donor blood to replace erythrocytes and sustain hematocrit during clamping procedures, which results in larger colony number and more complex experimental design (137). Independently of rodent model choice, however, blood glucose assessments must take into consideration; strain (40, 139), age (140, 141), sex (142–144), fasting length (137, 145) and husbandry (146–148) as all of these parameters differentially impact glucose homeostasis and the translatability of each model to human physiology (discussed in detail in 7,9). Non-mammalian species, while having critical distinctions in their mechanisms of glucose homeostasis, particularly with respect to the brain, offer unique opportunities afforded by their genetic tractability, lower cost, and fecundity. In particular, zebrafish offer a powerful platform for genetic manipulation, live imaging, neural recording, and high throughput screening with some comparable neuroendocrine processes to mammals. Ultimately, the use of model organisms permits investigation into brain-body interactions underlying glucose homeostasis with a level of detail not achievable using studies in humans.

## Author Contributions

All authors contributed to the article and approved the submitted version.

## Funding

This work was support by grants from Diabetes UK (19/0006035 to KE and CB, which funds AM) and the Juvenile Diabetes Research Foundation (1-INO-2020-919-A-N to CB which funds AC). YY is supported by Expanding Excellence in England.

## Conflict of Interest

The authors declare that the research was conducted in the absence of any commercial or financial relationships that could be construed as a potential conflict of interest.
